# Revealing the Crucial Role of Healthcare Providers in HPV Vaccine Uptake in Filipino American Adolescents: Insights from the National Immunization Survey-Teen, 2015–2019

**DOI:** 10.1007/s40615-025-02749-3

**Published:** 2025-12-02

**Authors:** Alexandra Marie Reyes, Elaine Zhiqing Liu, Philip T. Siu, Chun Pan, Grace X. Ma, Lin Zhu

**Affiliations:** 1 Center for Asian Health, Lewis Katz School of Medicine, Temple University, Philadelphia, PA, USA; 2 Esperanza Health Center, Philadelphia, PA, USA; 3 Department of Mathematics and Statistics, Hunter College, College University of New York, New York, NY, USA

**Keywords:** Human papillomavirus vaccine, HPV vaccine, Filipino american, Disaggregated data, Multilevel factors

## Abstract

**Background:**

HPV vaccines are highly effective in preventing cervical, anal, and several other pre-malignant and malignant disease caused by vaccine-specific HPV types. Despite increasing public health efforts in the past decade, HPV vaccination rates among Filipino American (FA) adolescents remain suboptimal. Our knowledge is particularly limited on how provider or practice-level characteristics were linked to uptake in this population, partially due to lack of disaggregated data.

**Methods:**

This study analyzed a sample of 669 FA adolescents, aged 13–17, using data from the National Immunization Survey-Teen (2015–2019). The aim was to identify risk factors affecting HPV vaccine uptake and completion in FA adolescents, and to compare these findings with those of an aggregate Asian American sample. Survey-weighted multivariate logistic regression was conducted to examine multilevel factors associated with vaccination.

**Results:**

Slightly over two-thirds (69.02%) of FA adolescents received at least one dose of the HPV vaccine, but only about one-third (37.65%) completed the regimen. About three quarters (74.16%) reported receiving a recommendation from their provider for the HPV vaccine, and 70.97% reported having a pediatrician. Logistic regression results showed that both provider’s recommendation and having a pediatrician were significant predictors of higher likelihoods of initiation and completion of HPV vaccine series (*p* < .05).

**Conclusion:**

The results of this study provide implications for improving clinical practices to promote HPV vaccine uptake in FA adolescents. Strategies to consider include raising awareness, cultural competency, and communication approaches among physicians, especially non-pediatricians. In addition, our findings highlighted the importance of disaggregating Asian American data to better understand the unique needs of detailed Asian ethnic groups.

## Introduction

Human papillomavirus (HPV) is one of the most common sexually transmitted infections in the United States, with an estimated 43 million cases reported in 2018 [1]. Certain strains of HPV can cause warts, and while others are linked to different types of cancer, including those of the cervix, vagina, vulva, penis, anus, and oropharynx. HPV vaccination can prevent over 90% of cancers caused by HPV, as well as vaginal, cervical, anal, and vulvar precancers [2]. Among all racial groups, Asian Americans (AAs) aged 9–26 have the lowest level of HPV vaccination initiation [3, 4]. However, there is a lack of disaggregated data regarding HPV vaccine uptake among Asian American and Native Hawaiian/Pacific Islander (AANHPI) groups. Additionally, Filipino Americans (FAs) are disproportionately affected by cervical, colorectal, and prostate cancer, which are common types of cancer in this population [4–7]. Data from the Surveillance, Epidemiology, and End Results Detailed Asian/Pacific Islander database showed that Filipino American women had a significantly higher risk for HPV-associated cancers, compared to their non-Hispanic white counterparts [4], indicating the need for improved HPV vaccination outreach and preventative care.

Unfortunately, our knowledge of HPV vaccine uptake among FAs is limited. While one multi-ethnic study in Hawaii found that lower HPV vaccination rates were reported by Filipino American parents than Caucasian parents, a more recent study using the National Health Interview Survey found no significant differences in HPV vaccine rates between Filipino and non-Hispanic white adults [8]. The latter study also noted substantial variations in HPV vaccination rates and trends across Asian ethnic groups, highlighting the need for research that details the unique patterns and associated factors of HPV vaccine in each Asian ethnic group separately [8].

Predictors of HPV vaccine uptake in FAs are not yet well understood. Existing literature shows that in the Philippines, HPV vaccine uptake was highly dependent on cost and insurance coverage [9, 10], but it is unclear if these factors are as influential for Filipinos living in the U.S. U.S. studies show that physicians were parents’ primary source of information about the HPV vaccine, but many parents remain unaware of specific recommendations and timelines for vaccination [11, 12]. A multi-ethnic study in Hawaii found that Filipino parents were significantly less likely to vaccinate their children compared to the other racial/ethnic groups [12]. The finding further suggested that FA parents, especially those with limited English proficiency, may have clinical experiences with physicians that are perceived as different or less convincing compared to those of other Asian ethnic groups [12]. Nonetheless, receiving a physician’s recommendation and the desire to protect their children from cancer were significant motivators for FA parents to have their children vaccinated against HPV [12]. Having friends whose children were vaccinated and having a generally positive attitude towards all vaccines are also mentioned as reasons why they opted for the HPV vaccine for their children [12].

FA parents who did not vaccinate their children against HPV often did not do so due to a lack of knowledge about the vaccine, concerns about its safety, and the belief that their children were not sexually active [12]. Additionally, a study of COVID-19 vaccine decision-making in FAs highlighted how limited English proficiency, being unfamiliar with the US healthcare and insurance system, potential side effects, and fear of political influences contributed to general vaccine hesitancy [13]. Furthermore, research on other populations has suggested several provider- and practice level factors such as physician specialty, practice facility type, and vaccine acquisition source, as significant predictors of HPV vaccine [14–16]. Whether these factors impact HPV vaccine uptake in Filipino Americans remains to be examined.

To the best of our knowledge, this study is the first to use a nationally representative sample of Filipino American adolescents between the ages of 13 and 17 to examine how various adolescent-, parent-, provider-, and practice-level factors are associated with HPV vaccination initiation and completion. Understanding the patterns of HPV vaccine uptake among this population and identifying facilitators and barriers to HPV vaccination can inform public health policies and clinical practices to develop targeted and culturally appropriate interventions to improve HPV vaccine awareness and uptake, and ultimately reduce HPV-related cancer burden in this population.

## Methods

### Data Source

This study utilized data from 2010 to 2019 of the National Immunization Survey-Teen (NIS-Teen), a continuous cross-sectional survey supported by the CDC and conducted by the National Opinion Research Center (NORC). NIS-Teen, conducted via random-digit-dial telephone interviews, collects demographic information and HPV vaccination status from parents or guardians of adolescents aged 13–17. The household surveys collect data through telephone interviews with parents or guardians in all 50 states, the District of Columbia, and some U.S. territories. Cell phone numbers are randomly selected and called to enroll one or more age-eligible teens from the household. The parents and guardians of eligible teens are asked during the interview for the names of their children’s vaccination providers and permission to contact them. Subsequently, with verbal consent from parents or guardian, a questionnaire is sent via mail to the adolescents’ immunization providers to request information on the types of vaccinations, number of doses, dates of administration, and other administrative data about the health care facility [17]. For brevity, we’ll refer to the parent or guardian interviewed as the adolescent’s parent. Population-level weights are developed to account for the multi-level sampling design used. The methods and weighting procedures for NIS-Teen were described previously [18]. This study included only 669 adolescents who were identified as FAs and for whom adequate provider data were available, ensuring sufficient vaccination information to determine their vaccination status. The aggregate AA sample included all teens who were identified as Asian Indians, Chinese, Filipino, Japanese, Korean, Vietnamese, or other Asians.

### Measures

#### HPV Vaccine Initiation and Completion

We used data in the provider survey to define HPV vaccine uptake status. Specifically, HPV vaccination initiation (yes/no) was defined as having received at least one dose of HPV vaccine, according to the provider-reported number of HPV vaccine doses adolescents received by the time of the interview. HPV vaccination completion (yes/no) was defined as receiving two doses if initiated before the age of 15, or three doses if initiated at or after the age of 15 [1].

#### Provider- and Practice-level Factors

Two provider-level factors were examined, including whether provider recommended HPV vaccine to adolescents (yes/no) and the provider’s specialty in six categories (pediatrics, family practice, general practice, internal medicine, OB/GYN, or other). Three practice-level factors were included in this study. Firstly, the types of healthcare facilities were categorized into six groups: all public, all private, all hospital facilities, all specialized clinics (e.g., STD/school/teen clinics), mixed, or other. Secondly, vaccine acquisition source was determined by whether the practices ordered HPV vaccines from the state/local health department through the Vaccine for Children (VFC) program (yes/no). Lastly, practices reported whether they conducted the 11–12-year-old well child exam or check-up for adolescents (yes/no).

#### Parent-level Factors

We examined two sociodemographic characteristics of the parents from the household survey, including the mother’s educational level, measured in four categories (< 12 years, 12 years, >12 years non-college graduate, and college graduate) and mother’s marital status in two categories (currently married and not currently married).

#### Adolescent-level Factors

We also examined seven characteristics of adolescents from the household survey. Sex was measured in two categories, male or female. The census region of the residence was measured in four categories (Northeast, Midwest, South, and West). The current grade of the adolescents was measured in three categories (6th to 8th, 9th to 12th, and high school [HS]/general educational development [GED]/not in school). The health insurance status was measured in four categories (private insurance only, any Medicaid, other insurance, and uninsured). Household poverty level was measured in three categories (above poverty level and >$75k, above poverty level <= $75k, and below poverty level). Nativity status was measured in two categories, whether the adolescent was born in the US or not.

### Statistical Analysis

Following the instructions in the NIS-Teen User’s Guide [19], we used the appropriate weights and stratum variables to weigh the sampled individuals to be representative of the demographics of the U.S. population. The adjustment accounted for the non-response, no-resolution of telephone numbers, missing provider data, and the complex survey design. For calculating average estimates over the years, the NIS-Teen data from multiple years are combined, requiring the annual weights to be divided by the number of years amalgamated [19]. All analyses conducted were weighted. We presented the weighted descriptive statistics, including percent and 95% confidence interval (CI) of various adolescent-, parent-, provider-, and practice-level characteristics of the Filipino American sample, and for comparison, those of the aggregate Asian sample. To examine the multilevel factors associated with HPV vaccine initiation and completion, we conducted survey-weighted multivariate logistic regression. The odds ratio (ORs) with 95% CI were presented. The statistical analysis was performed using Stata version 16 [20]. We used the *svy* function in Stata to apply weighting, and the *subpop* option for subpopulation analyses. A *p*-value < 0.05 was considered statistically significant.

## Results

The weighted socioeconomic and immigration-related characteristics of the adolescents and parents of the FA adolescents and the aggregate Asian American adolescent sample are presented in Table 1. Among the study sample, the mean age was 15.04 years, and consisted of 53.35% boys and 46.65% girls. About one-third of the adolescents (34.85%) had a family income of $ 75 K or less, with 4.63% without any health insurance. Moreover, 15.08% of FA adolescents were born outside of the US. With regards to the parental socioeconomic status, 19.16% of the FA adolescents reported that their mothers were not married at the time of the survey, and about half (53.89%) had mothers with a college degree. The FA sample varied significantly from the aggregate AA sample in several characteristics. The majority of the FA adolescents resided in the West (53.55%), while the AA adolescents were more evenly distributed across regions. The proportion of FA adolescents in 9th to 12th grade (68.00%) was lower than that of the AA adolescents (72.25%). FA adolescents were more concentrated in the West, had a lower proportion in higher grade levels, and had both a higher proportion of private insurance coverage and household incomes above $75,000 compared to AA adolescents. Conversely, AA adolescents had a higher proportion of Medicaid coverage than FA adolescents.

Table 2 presents the weighted provider- and practice-level characteristics for the FA sample and the aggregated Asian American sample. Among FA adolescents, 74.16% received a recommendation for the HPV vaccine from their providers. About half 53.55% reported hospitals as the only types of facilities where their providers were located. The majority (70.97%) of the FA adolescents had a pediatrician, 32.74% had a family practitioner, while 21.47% had an internalist. About four out of five (84.26%) in the FA sample had a provider who ordered vaccines from state or local health departments through the Vaccines for Children (VFC) Program. In addition, 92.00% of the FA adolescents reported receiving the 11–12-year-old well child exam from their providers. We noted significant variations between the FA and aggregate AA sample on several factors, including types of facility and provider specialty. Specifically, FA adolescents had significantly less access to providers from private facilities and hospital facilities, and more frequently received care from general and OB/GYN practitioners, compared to the aggregated AA adolescents.

**Fig. 1:**
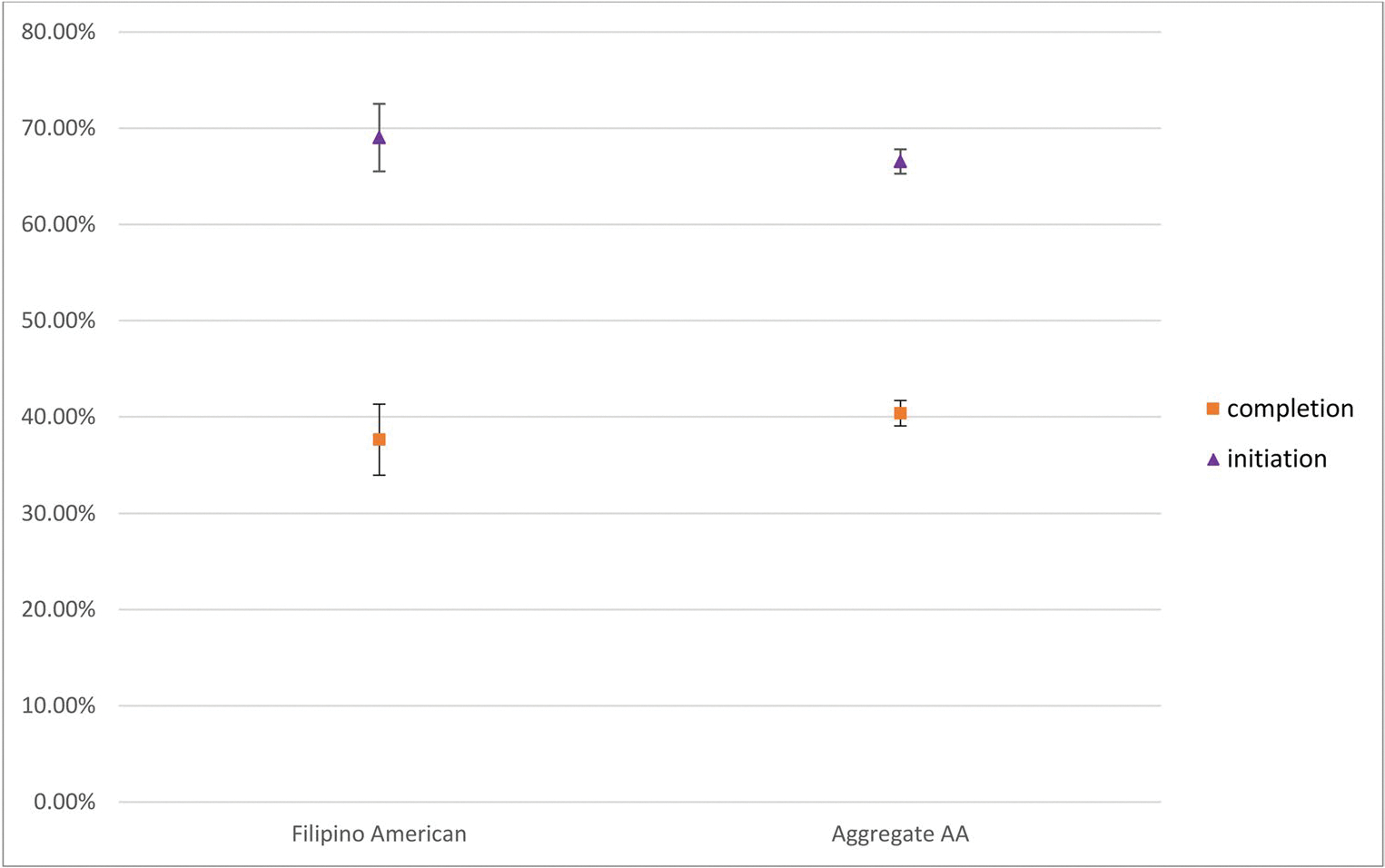
Provider reported HPV vaccine initiation rate in the Filipino American and aggregate Asian American samples

Table 3; Fig. 1 present the weighted HPV vaccination initiation and completion rates, as recorded in the providers’ records. Based on the provider reports, only about two thirds (69.02%) of the FA adolescents received at least one shot, while about one third (37.65%) completed their HPV vaccine regimen. Vaccine uptake was even lower according to household-reported data with only half (51.52%) initiating the vaccine and one in five (21.85%) completing the HPV vaccine regimen (results not included). In addition, “not receiving a recommendation from the provider” was cited by 18.56% of the unvaccinated FA adolescents as a reason for not receiving any HPV vaccines. This was much lower than the 24.19% rate in the aggregated AA adolescents. Other reasons were examined, but the results were not presented because of low case counts, in compliance with the NIS-Teen data release policy.

Table 4 presents the results of the multivariate logistic regression on HPV vaccination initiation. We found that receiving a physician’s recommendation for the HPV vaccine (OR = 3.07, 95%CI = 1.27–7.42) was significantly associated with a higher likelihood of initiating the HPV vaccine regimen. Having a pediatrician (OR = 2.99, 95%CI = 0.86–10.34.86.34) was a marginally significant predictor of HPV vaccination initiation. In addition, our results showed that FA adolescents living in the West was more likely than those living in the South to initiate HPV vaccination (OR = 4.32, 95%CI = 1.70–11.00). Surprisingly, FA girls were less likely than boys to initiate HPV vaccination (OR = 0.51, 95%CI = 0.23–1.14).

Table 5 presents the association of various factors with HPV vaccination completion in FA adolescents. Having a provider recommendation (OR = 4.58, 95% CI = 1.35–15.56) and having a pediatrician (OR = 5.06, 95% CI = 1.71–15.00) were both significantly associated with a higher likelihood of completing the HPV vaccine regimen. Additionally, FA girls were marginally more likely than boys to complete the HPV vaccine regimen (OR = 2.19, 95%CI = 0.97–4.91).

## Discussion

To the best of our knowledge, this is the first study to employ a nationally representative sample to examine HPV vaccine uptake among Filipino Americans, a population with unique socioeconomic and health characteristics. Our findings offer crucial epidemiological data for this population, laying the groundwork for public health initiatives and informing social policy decisions related to vaccine promotion. Specifically, our investigation revealed suboptimal HPV vaccine uptake among Filipino American adolescents, with only two-thirds (69.02%) of the cohort receiving at least one vaccination dose and merely one-third (37.65%) completing the vaccine regimen.

In comparison, the 2021 NIS-Teen data revealed an 82.8% HPV vaccine initiation rate and a 72.3% completion rate among non-Hispanic Asian adolescents [21]. While these uptake rates appear to approach the 80% HPV vaccination benchmark established by the Healthy People 2023 initiative [22], they must be interpreted with caution and nuance. A cross-sectional analysis of recent data from the 2020–2022 NIS-Teen survey revealed that HPV vaccination initiation did not increase among adolescents for the first time since 2013, a stagnation likely due to missed well-child appointments and increased vaccine hesitancy resulting from the COVID-19 pandemic [23]. Notably, there was a decrease in HPV vaccine initiation among adolescents eligible for the Vaccines for Children program, particularly among those insured by Medicaid and uninsured [23]. This group constituted approximately 20% of the FA sample and 27% of the aggregate AA sample in our study [23]. This decline demonstrates an urgent need for resources to support catch-up vaccination, especially among the adolescents eligible for VFC and in other underprivileged demographic groups. Immediate actions are needed to implement culturally appropriate outreach and interventions tailored aimed at increasing vaccine uptake in these communities. These efforts should prioritize raising awareness, address concerns voiced by adolescents and parents, and improve access to healthcare services. For example, community-based interventions could include multilingual educational campaigns delivered through trusted community organizations, clinical partners, patient navigation programs to address vaccine safety concerns and scheduling barriers, and school-located vaccination clinics to reduce transportation and time-related obstacles. Considerations should also be made to address general vaccine hesitancy that was impacted by the COVID-19 vaccine hesitancy and the public mistrust in public health and medical authorities related to the COVID-19 pandemic.

It is noteworthy that CDC updated HPV vaccination recommendation regarding dosing schedule in 2016, where 2 doses of HPV vaccine are recommended for people starting the vaccination series before their 15th birthday [24]. The 2-dose regimen entailing fewer visits could improve HPV vaccination completion rate while reducing total costs of HPV vaccination [25]. More research is needed to examine the 2-dose and 3-dose regimen completion rate in the respective cohorts in Filipino American adolescents and other Asian ethnic groups. Such information would help provide epidemiological evidence for targeted outreach and promotional efforts.

Consistent with previous research [24–26], our results emphasized the crucial role of healthcare providers play in HPV vaccine uptake, with insights specifically relevant to Filipino American adolescents. Our findings indicate that provider recommendations are essential to both the initiation and completion of HPV vaccination series. This indicates the need for continued outreach and training for healthcare providers to promote the HPV vaccine effectively. Previous studies have found specific communication strategies such as the use of presumptive language and strong recommendation strategies tailored to patient’s age group, as effective in improving HPV vaccine uptake in the general population [27, 28]. How these strategies could be adapted for the FA community remains to be fully explored.

Furthermore, provider specialty was an important factor; having access to a pediatrician was a marginally significant predictor of higher HPV vaccine uptake, which is consistent with previous literature [29–32]. In contrast, access to other specialties, such as family practice, general practice, or obstetrics and gynecology was not significantly associated with HPV vaccine uptake. The variations in HPV vaccine uptake based on provider specialty highlight important implications. Given the significant rise in adolescents seeing family physicians in recent decades [33], it is crucial that family physicians and general practitioners improve their strategies to reduce missed clinical opportunities and improve HPV vaccine delivery. Strategies should include outreach to underserved communities, language assistance to patients and families with limited English proficiency, and culturally tailored communication to engage patients in meaningful and effective vaccine discussions.

Two additional factors were examined at the practice- or systemic-level: the ability of healthcare practices to order vaccines from state or local health departments and whether a 11–12-year-old well child exam was conducted. However, neither factor was found to be significantly associated with HPV vaccine uptake among Filipino American adolescents. While the VFC program offers free vaccination to uninsured, low-income, and Medicaid-eligible adolescent patients through state health departments, the impacts of this program in HPV vaccine uptake remains unclear [34, 35]. Were eligible adolescents and families made aware of the VFC program? What were some of the facilitators and barriers to establishing systems for ordering HPV vaccines through the health department? What efforts were involved in stocking HPV vaccines, managing personnel or ordering and inventory, and receiving reimbursement for the VFC-enrolled practices? Building on our previous discussion, how do these factors impact providers across different specialties? The answers to these questions will provide insight into how the VFC program and operational factors at the organization- or systemic-level directly impact HPV vaccine uptake [36].

One surprising and noteworthy finding is that while girls were more likely than boys to complete the HPV vaccine regimen, they were less likely than boys to initiate the vaccine. This reversal of patterns was not observed in the aggregate Asian American sample, where girls were more likely than boys to initiate and complete the HPV vaccine regimen in the adjusted regression models (results available upon request). One potential explanation to the gender differences in vaccine initiation and completion among the FA sample is the “head start” girls had on boys. When the HPV vaccine was introduced in 2006 [24], it was primarily marketed towards and recommended for girls, with the primary focus on cervical cancer prevention. While the United States Food and Drug Administration (FDA) approved its use for boys in 2009 [37], the CDC did not officially recommend routine HPV vaccination for boys until 2011 [24]. This could explain the notable increase in vaccine uptake among boys in more recent years compared to earlier periods [38, 39]. The higher initiation rate among FA boys compared to girls observed in our study may reflect several factors that warrant further investigation. Targeted catch-up vaccination efforts and intensified healthcare provider recommendations for adolescent boys following the 2011 universal recommendation may have particularly resonated with FA families. Additionally, culturally specific factors within Filipino communities may contribute to differential vaccine acceptance by gender. Parents of FA girls may harbor greater concerns about HPV vaccination due to misconceptions linking the vaccine to earlier sexual debut or promiscuity, concerns that have been documented in other cultural contexts and may be particularly salient in communities with conservative norms around female sexuality [40, 41]. These potential explanations require dedicated research to understand the specific mechanisms driving this unique gender pattern in the FA population. The observed time lag in HPV vaccine completion among the boys in our study was likely due to their later initiation of vaccination compared to girls in the FA sample. While this finding of our study suggested largely positive vaccination trends, it underscores the need for more culturally tailored outreach and promotion efforts in FA communities. Considering unique cultural norms related to gender roles and sexual behaviors [42, 43], as well as immigration-related factors is essential to effectively engage FA adolescents and their families in the vaccine decision-making process.

Our study was not without limitations. Firstly, the cross-sectional nature of the data restricted our ability to make causal inferences. Longitudinal studies are necessary to comprehensively understand how factors at multiple levels interact over time to influence vaccine uptake outcomes. Secondly, the role of pharmacists in HPV vaccine delivery was not considered in the study. As confirmed in previous studies, local pharmacies and pharmacists play critical roles in healthcare deliveries in diverse Asian American communities [44–46]. Leveraging the extensive reach of community pharmacies, especially in racial/ethnic minority enclaves and medically underserved areas, has been identified as a promising strategy to increase HPV vaccine uptake [47, 48]. Future research may explore how local pharmacies can facilitate HPV vaccine awareness and uptake in FA adolescents.

The findings of this study shed light on the need for culturally appropriate public health and clinical efforts in improving HPV vaccine delivery to FA adolescents. These efforts must consider factors at both the adolescent and parent level, as well as within the provider and practice system. Involving FA adolescents, their families, community stakeholders, and healthcare providers is critical to ensure that the intervention efforts are truly accessible and culturally appropriate for the target population.

The significance of this study extends beyond the FA community. It highlights disparities observed between FA and aggregate AA samples across various sociodemographic factors, healthcare access and utilization patterns, as well as HPV vaccine uptake. Grouping diverse Asian ethnic groups together can obscure different disease burdens and needs within each population, hindering efforts to improve healthcare access and outcomes for these communities [49]. By acknowledging both the commonalities and differences across various Asian subpopulations, researchers, healthcare providers, educators, and policymakers can gain a deeper understanding of the communities they serve and develop effective strategies to improve health outcomes for all [50].

## Figures and Tables

**Table 1:** Weighted descriptive statistics of Adolescent- and Parent-Level sociodemographic and Immigration-Related characteristics (NIS-Teen 2015–2019)

Proportion or mean (95% CI)	FA (*N* = 669)	Aggregate AA (*N* = 5,321)

Age (mean)	15.04363 (14.7692, 15.1103)	14.99 (14.9116, 15.0684)
Sex		
Male	0.5335 (0.4957, 0.57137)	0.4971 (0.4837, 0.5105)
Female	0.4665 (0.42870, 0.5043)	0.5029 (0.4837, 0.5105)
Census region of residence		
Northeast	0.0853 (0.0641, 0.1064)	0.2052 (0.1943, 0.2161)
Midwest	0.1186 (0.0941, 0.14310)	0.1552 (0.1455, 0.1649)
South	0.2606 (0.2273, 0.2939)	0.2778 (0.2658, 0.2898)
West	0.5355 (0.4977, 0.5733)	0.3619 (0.3490, 0.3748)
Current grade		
6th – 8th	0.3186 (0.2833, 0.3539)	0.2655 (0.2536, 0.2774)
9th to 12th	0.6800 (0.6447, 0.71535)^a^	0.7225 (0.7105, 0.7345)
HG/GED/not in school	-	0.0121 (0.0092, 0.0150)
Insurance		
Private insurance only	0.6857 (0.6505, 0.7209)	0.6509 (0.6381, 0.6637)
Any Medicaid	0.1523 (0.1251, 0.1795)	0.2383 (0.2269, 0.2497)
Other insurance	0.1156 (0.09137, 0.1398)	0.0726 (0.0656, 0.0796)
Uninsured	0.0463 (0.03038, 0.0622)	0.0382 (0.0330, 0.0434)
Household income poverty level		
Above poverty >$75k	0.6515 (0.6154, 0.6876)	0.5929 (0.5797, 0.6061)
Above poverty <= $75k	0.2655 (0.2320, 0.2990)	0.2575 (0.2458, 0.2692)
Below poverty	0.083 (0.0621, 0.1039)	0.1496 (0.1400, 0.1592)
Nativity status		
US-born	0.8492 (0.8221, 0.8763)	0.8119 (0.8014, 0.8224)
Foreign-born	0.1508 (0.1237, 0.1779)	0.1881 (0.1776, 0.1986)
Marital status of mother		
Married	0.8084 (0.7786, 0.8382)	0.805 (0.7944, 0.8156)
Not currently married	0.1916 (0.1618, 0.2214)	0.195 (0.1844, 0.2056)
Education of mother		
< 12 years	0.0099 (0.0024, 0.0174)	0.0897 (0.0820, 0.0974)
12 years	0.1458 (0.1191, 0.1725)	0.1565 (0.1467, 0.1662)
>12 years, non-college grad	0.3054 (0.2705, 0.3403)	0.1325 (0.1234, 0.1416)
College graduate	0.5389 (0.5011, 0.5767)	0.6214 (0.6084, 0.6344)

a Results of “9th to 12th” and “HG/GED/not in school” combined because of low case count in “HG/GED/not in school”

**Table 2:** Weighted percentage (95% CI) of Provider- and Practice-Level characteristics (NIS-Teen 2015–2019)

Proportion or mean (95% CI)	FA (*N* = 669)	Aggregate AA (*N* = 5,321)

Provider recommendation for HPV vaccine		
Yes	0.7416 (0.7124, 0.7708)	0.7309 (0.7190, 0.7428)
No	0.2584 (0.2292, 0.2876)	0.2691 (0.2572, 0.2810)
Type of facility for teens providers		
All public facilities	0.1204 (0.1069, 0.1339)	0.1003 (0.0922, 0.1084)
All private facilities	0.0517 (0.0292, 0.0742)	0.0874 (0.0798, 0.0950)
All hospital facilities	0.5355 (0.4993, 0.5717)	0.6394 (0.6265, 0.6523)
All STD/school/teen clinics or other facilities	0.0531 (0.0358, 0.07038)	0.0391 (0.0339, 0.0443)
Mixed	0.2393 (0.2109, 0.2677)	0.1338 (0.1247, 0.1429)
Other		
Specialty of provider		
Pediatrics	0.7097 (0.6804, 0.7390)	0.7454 (0.7337, 0.7571)
Family practice	0.3274 (0.29235, 0.3624)	0.2928 (0.2806, 0.3050)
General practice	0.1821 (0.15590, 0.2083)	0.1371 (0.1279, 0.1463)
Internal medicine	0.2147 (0.1836, 0.2458)	0.1736 (0.1634, 0.1838)
OB/GYN	0.1887 (0.1627, 0.2147)	0.1291 (0.1201, 0.1381)
Other	0.1935 (0.1626, 0.2244)	0.1540 (0.1443, 0.1637)
Whether provider ordered vaccines from state/local health dept		
Some or all	0.8426 (0.8103, 0.8749)	0.8188 (0.8085, 0.8292)
No	0.1574 (0.1251, 0.1897)	0.1812 (0.1709, 0.1916)
Whether provider conducted 11–12-year-old well child exam to teen		
Yes	0.92 (0.9117, 0.9283)	0.9049 (0.8970, 0.9128)
No	0.08 (0.0717, 0.0883)	0.0951 (0.0872, 0.1030)

**Table 3:** Weighted statistics of HPV vaccination initiation and completion Rates, and reasons for not receiving HPV vaccines (NIS-Teen 2015–2019)

Proportion or mean (95% CI)	FA (*N* = 669)	Aggregate AA (*N* = 5,321)

HPV vaccination initiated		
Yes	0.6902 (0.6551, 0.7252)	0.6654 (0.6527, 0.6781)
No	0.3098 (0.2748, 0.3448)	0.3346 (0.3219, 0.3473)
HPV vaccination completed		
Yes	0.3765 (0.3398, 0.4132)	0.4037 (0.3905, 0.4169)
No	0.6235 (0.5868, 0.6602)	0.5963 (0.5831, 0.6095)
Reasons for not receiving HPV vaccines		
Not recommended	0.1856 (0.1561, 0.2151)	0.2419 (0.2304, 0.2534)

**Table 4:** Logistic regression results of provider reported HPV initiation in FA Adolescents, odds ratio and 95% confidence interval (NIS-Teen 2015–2019)

	Odds Ratio (95%CI), *p*-value

*Adolescent-level characteristics*	
Sex	
Male	Reference
Female	0.5106387 (0.2286, 1.1407), 0.010
Census region of residence	
Northeast	1.495166 (0.4440, 5.0354), 0.516
Midwest	2.175491 (0.71702, 6.6006), 0.170
South	Reference
West	4.31924 (1.6962, 10.9987), 002
Insurance	
Private insurance only	0.3811 (0.0469042, 3.0970), 0.367
Any Medicaid	2.1818 (0.2333, 20.4081), 0.494
Other insurance	0.1636 (0.0177, 1.5116), 0.111
Uninsured	Reference
Nativity status	
US-born	Reference
Foreign-born	0.5744 (0.2029, 1.6263), 0.296
*Provider-/practice-level characteristics*	
Provider recommendation of HPV vaccine (ref: no)	3.0686 (1.2697, 7.4166), 0.013
Pediatrics specialty (ref: no)	2.9895 (0.8641, 10.3420), 0.084
Provider ordered vaccines from state/local health dept (ref: no)	1.4966 (0.4836, 4.6314), 0.484

**Table 5:** Logistic regression results of provider reported HPV completion in FA Adolescents, odds ratio and 95% confidence Interval (NIS-Teen 2015–2019)

Adolescent-level characteristics	Odds Ratio (95%CI), *p*-value

Sex	
Male	Reference
Female	2.1899 (0.9772, 4.9072), 0.057
Current grade	
6th – 8th	0.1287 (0.0031, 5.4238), 0.283
9th to 12th	0.3835 (0.0093, 15.8205), 0.614
HG/GED	Omitted
Not in school	Reference
Insurance	
Private insurance only	1.192685 (0.1644, 8.6509), 0.862
Any Medicaid	1.3044 (0.1413, 12.0427), 0.815
Other insurance	0.1920 (0.0178, 2.0758), 0.174
Uninsured	Reference
*Parent-level characteristics*	
Education of mother	
< 12 years	1.9422 (0.3444, 10.9521), 0.452
12 years	1.7011 (0.4122, 7.0197), 0.463
>12 years, non-college grad	1.7044 (0.5915, 4.9117), 0.323
College graduate	Reference
*Provider/practice-level characteristics*	
Provider recommendation of HPV vaccine (ref: no)	4.5785 (1.3471, 15.5607), 0.015
Pediatrics specialty (ref: no)	5.0580 (1.7057, 14.9985), 0.003
Provider ordered vaccines from state/local health dept (ref: no)	1.1974 (0.4323, 3.3170), 0.729
Provider conducted 11–12-year-old well child exam to teen	0.7164 (0.0517, 9.9192), 0.804

* *p*<.05

** *p*<.01

*** *p*<.001

## Data Availability

The NIS-Teens data are available at the CDC Website: https://www.cdc.gov/nchs/nis/data_files_teen.htm.
